# Dietary Inclusion of 1,3-Butanediol Increases Dam Circulating Ketones and Increases Progeny Birth Weight

**DOI:** 10.3390/ani9080479

**Published:** 2019-07-24

**Authors:** Udani Wijesiriwardana, John R. Pluske, Jessica R. Craig, Jeremy Cottrell, Frank R. Dunshea

**Affiliations:** 1 Faculty of Veterinary and Agricultural Sciences, The University of Melbourne, Parkville, VIC 3010, Australia; 2 Agricultural Sciences, College of Science, Health, Engineering and Education, Murdoch University, Murdoch, WA 6150, Australia; 3 Australasian Pork Research Institute Ltd. (APRIL), Willaston, SA 5118, Australia; 4 Research and Innovation, Rivalea (Australia) Pty. Ltd., Corowa, NSW 2646, Australia

**Keywords:** gilt progeny, parity, birthweight, ketones, pre-weaning mortality

## Abstract

**Simple Summary:**

Gilt progeny are born and weaned lighter and have poorer life-time performance than sow progeny. Low birth weights and pre-weaning mortality are highly associated with and are often a result of reduced milk consumption and vigor. Glycogen stores are deposited in utero and are relied on heavily within the first hours of life. Because of rapid depletion of these stores, piglets must consume enough milk immediately post-partum for survival. Similar to glucose, ketone bodies have the ability to readily pass the placenta for the piglet to use in the neonatal period. Supplementing late gestation diets with ketogenic substances as an alternative source of energy can potentially be used by the piglet, reducing the rapid depletion of glycogen stores. This study supplemented late gestation diets of both gilts and sows with the ketogenic substance 1,3-Butanediol and found that birth weights and total litter weights were increased in both gilt and sow progeny. While Butanediol can potentially increase birth weight and growth performance in the post-parturition period, a strong parity effect was still evident throughout the study with sow progeny outperforming gilt progeny

**Abstract:**

1,3-Butanediol (BD) is a ketogenic substance that can improve piglet growth and survival and potentially increase performance in gilt progeny when provided as a dietary supplement during late gestation. Gilts (n = 77; parity 1) and sows (n = 74; parities 2 and 3) were fed either a standard commercial gestation diet or a diet supplemented with 4% BD from day 90 of gestation until farrowing. Dams fed with diets supplemented with BD had higher plasma beta-hydroxybutyrate (*p* = 0.01) and lower non-esterified fatty acid concentrations (*p* < 0.001). The percentage of progeny that were light-for-age (<1.1 kg) at birth was decreased by BD (18.2 vs. 13.5%, *p* < 0.006), particularly in gilts (24.0 vs. 18.3%, *p* < 0.034). Individual birth weights and litter weights birth weights tended to be increased by the BD diet (*p* = 0.085 and 0.078; respectively) although these effects were not maintained to weaning. Pre-weaning mortality was greater in gilt than in sow progeny and was not altered by dietary BD. Feeding BD in late gestation can improve birth weight, but further work is needed to see if these effects are carried through subsequent stages of growth, particularly in gilt progeny.

## 1. Introduction

Gilt progeny (GP) present a significant burden on pig producers since they exhibit lower birth and weaning weights, a higher risk of disease and mortality, and poorer whole-of-life performance compared to sow progeny (SP) [1]. Low birth weight (LBW) or light-for-age piglets, those between 0.8 and 1.1 kg [2], and pre-weaning mortality are strongly linked, since smaller piglets are born with lower body energy reserves in the form of glycogen and show reduced thermoregulatory capacity [3]. This in turn results in more time taken to reach the udder, and consequently, these piglets have reduced colostrum and milk consumption [4,5]. Furthermore, glycogen stores are rapidly depleted in the first 16 h of life, and therefore, energy supplied from colostrum is imperative to piglet survival [6].

Neonatal piglets are born devoid of body fat due to an inability of lipids to pass transplacentally from the sow to the fetus. Therefore, piglets rely on glycogen stores accumulated from glucose passed from mother to fetus as a primary source of energy in the neonatal period, prior to colostrum consumption [7,8]. Ketone bodies are also readily able to pass through the placenta from mother to fetus, hence exogenous ketones offer a novel source of energy to the piglet and spares metabolism of glycogen stores deposited in utero in times of fasting or reduced nutrient intake. The neonatal period of life is a critical time for a piglet and can be a major cause of pre-weaning loss [9,10]. Therefore, formulation of gestation diets that can increase neonatal survival and reduce pre-weaning mortality are imperative for optimizing GP growth performance.

In this study, a late gestation diet supplemented with 1,3-Butanediol (BD), a synthetic carbohydrate which is metabolized to beta-hydroxybutyrate (β-OHB), was fed to gilts and sows. The aim of this study was to evaluate whether BD would increase birth weights and decrease pre-weaning mortality in GP by increasing circulating ketones (i.e., β-OHB) in gestating gilts and sows. The hypothesis examined was that progeny from dams fed with BD would have piglets with higher birth weights, fewer light-for-age piglets, and decreased pre-weaning mortality, with the responses being greater for progeny from gilts than from sows.

## 2. Materials and Methods

### 2.1. Ethics Statement

Experimental procedures were approved by the Rivalea (Corowa, Australia) Pty Ltd. Animal Care and Ethics Committee (protocol number 16P054C) in accordance with the Australian Code for the Care and Use of Animals for Scientific Purposes (National Health and Medical Research Council, 2013).

### 2.2. Animals, Diet and Experimental Design

The experiment was conducted under commercial conditions at a piggery in New South Wales, Australia (Rivalea Australia, Pty Ltd., Corowa, NSW, Australia), from June 2016 to February 2017. The experiment was a 2 × 2 factorial design (parity × diet) with a total of 77 gilts (parity 1) and 74 multiparous sows (parities 2, 3, and 4) being used across a total of five replicates. Gilts and sows were randomly allotted to their control or BD diet. From day 90 (90 ± 0.2 days; mean ± SE) gilts and sows were fed a common gestation diet (13.8 MJ digestible energy (DE)/kg, 14.3% crude protein (CP), 0.4% standardized ileal digestible (SID) Lys; as-fed basis) with experimental diets containing 4% BD replacing wheat (Table 1). Feed was delivered once daily at 09:00, with gilts and sows receiving 2.5 kg/day respectively from day 90 until moving to the farrowing house on day 110. Once in the farrowing house, both gilts and sows were fed 2.5 kg/day of their allotted diet until parturition, with all feed refusals recorded. Once farrowed, administration of the treatment diet ceased, and farrowed sows were placed on the commercial lactation diet at 2.5 kg on the day of farrowing, 3 kg one day post farrowing, and up to 4 kg from the second day having ad libitum access till weaning.

Gilts and sows were weighed and P2 backfat was measured at the P2 site, 65 mm down the side at the level of the head of the last rib. Weights and P2 backfat measurements were taken on day 90 of gestation and they were subsequently housed in groups of 7 depending on parity and diet from day 90 to day 110. Prior to farrowing room entry on day 110, gilts and sows were weighed again, and blood samples were taken. From day 110, gilts and sows were housed in the same farrowing room in alternating farrowing crates fitted with the nipple drinkers for mothers and piglets, and a heat lamp in creep area. All live piglets from each litter were individually weighed and given a numbered ear tag within 24 h of birth. This time was chosen for the initial weight so that there was no interference in piglets obtaining colostrum. Two focal piglets were selected for each litter for plasma samples within 24 h. Following this, cross-fostering was carried out following commercial procedures. Piglets were fostered within parity and diets when possible. The number of total piglets, number of piglets born alive, number of still births, and number of mummified fetuses were recorded for each litter. Piglets were individually weighed again at 21 days of age. A total of 1822 piglets were used for a birth weight measurement and 1518 piglets were used for 21-day weights. A total of 241 piglets were used for plasma metabolite analysis.

Blood from gilts, sows, and piglets was collected via jugular venipuncture into lithium and heparin Vacutainers (BD™ Vacutainer™, North Ryde, NSW, Australia) and inverted 4 times to ensure anticoagulant was distributed throughout the blood. Tubes were then placed on ice until centrifugation at 4 °C for 10 min. Plasma was collected and stored at −20 °C until analysis.

### 2.3. Metabolite and Hormone Analysis

Sow, gilt and piglet plasma was assayed for beta-hydroxybutyrate (β-OHB), non-esterified fatty acids (NEFA), glucose and insulin were assayed for gilts and sows only. Plasma β-OHB (Cayman Chemical, Ann Arbor, MI, USA; 0.7–16.2% intra-assay CV and 13.9% inter-assay CV), NEFA (NEFA-C, Wako Chemical Industries, Osaka, Japan; 0.5–10.7% intra-assay CV, 11% inter-assay CV), glucose (Thermo Fisher Scientific, Waltham, MA, USA; 1.3–7% intra-assay CV, 7.9% inter-assay CV), and insulin (Mercodia, Uppsala, Sweden; 0.5%–5.6% intra-assay CV, 10% inter-assay CV) concentrations were determined by following manufacturer’s procedures.

### 2.4. Statistical Analysis

Live weight, P2 backfat, metabolite data, and hormone data were analyzed by linear mixed model analysis using GENSTAT (16th edition). Parity and diet were analyzed as the main and interactive factors for all parameters. The replicate was designated as the random term for all production and metabolite analyses with sow ID designated as the random term for individual piglet growth and metabolite analysis. The percentage of light-for-age at birth (<1.1 kg) and pre-weaning mortality in piglets were analyzed using a Chi- Squared (χ^2^) test. Data are presented as a ± standard error of the differences (SED). A value of *p* < 0.05 was used to indicate statistical significance and a value of *p* < 0.10 was considered a statistical trend.

## 3. Results

### 3.1. Farrowing Performance

There was no effect of dietary BD on dam live weight just prior to farrowing (d110) (*p* = 0.11) or at weaning (*p* = 0.43) (Table 2). There was a tendency for dams on the control diet to have less P2 backfat compared to those on the BD diet (19.3 vs. 20.5 mm; respectively; *p* = 0.082) (Table 2). As expected, gilts were lighter just prior to farrowing (209 vs. 256 kg, *p* < 0.001) and at weaning (187 vs. 230 kg, *p* < 0.001). Sows tended to lose more live weight compared to gilts (−12.7 vs. −8.1 kg, *p* = 0.09) and lost more backfat (−3.3 vs. −2.0 mm, *p* = 0.019). There were no main or interactive effects of parity or dietary BD on total born alive, still born or mummified piglets (Table 2).

Piglets born to dams supplemented with BD tended to be 50 g heavier at birth compared to control dams (1.48 vs. 1.43 kg, *p* = 0.085), and these differences extended to total litter weights (17.9 vs. 17.0 kg, *p* = 0.078) (Table 2). Gilt progeny were 170 g lighter than sow progeny (1.37 vs. 1.54 kg, *p* < 0.001) at birth, and these differences also extended to total litter weight (16.3 vs. 18.6 kg, *p* < 0.001) (Table 3). Addition of BD tended to reduce individual piglet weights at 21 days (5.60 vs. 5.56 kg, *p* = 0.064) but this effect did not extend to total litter weights (56.8 vs. 56.7 kg, *p* = 0.83). However, there was an indication of an interaction (*p* = 0.10) that suggested that BD increased weaning litterweight of GP, whereas it was reduced in SP (Table 2). Gilt progeny were lighter at 21 days than SP (5.00 vs. 6.11 kg, *p* < 0.001), and these differences were evident in total litter weight (48.9 vs. 64.6 kg, *p* < 0.001) (Table 2). There were no effects of either BD or dam parity on the intra-litter coefficient of variation (CV) of birth or 21-day weights (Table 3).

**Figure 1:**
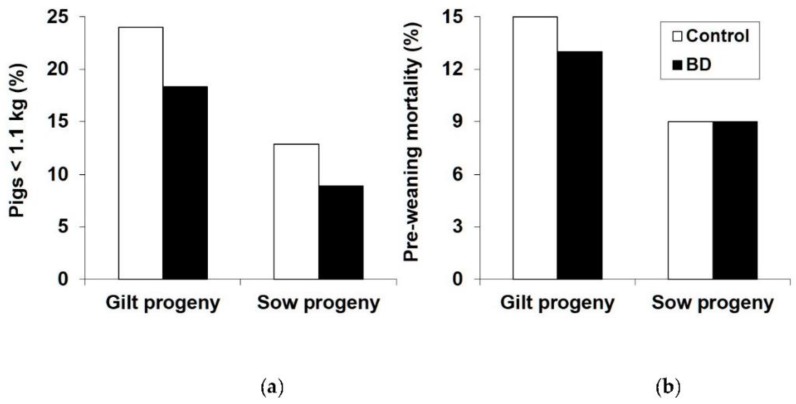
Effect of parity and diet (control vs. 1,3-Butanediol (BD) on: (**a**) Percentage of pigs that are small-for–age at birth (<1.1 kg); (**b**) Pre-weaning mortality. See text for statistical analysis.

The percentage of progeny that were light-for-age (<1.1 kg) at birth was decreased by BD (18.2 vs. 13.5%, *p* = 0.006), particularly in GP (24.0 vs. 18.3%, *p* = 0.034) (Figure 1a). Overall, SP had a lower percentage of progeny that were light-for-age at birth than GP (20.9 vs. 10.8%, *p* < 0.001) (Figure 1a). Pre-weaning mortality was lower in SP than in GP (8.83 vs. 13.7%, *p* < 0.001) but there was no effect of BD (11.6 vs. 10.9%, *p* = 0.64) (Figure 1b). Even within GP there was no significant effect of BD on mortality rate (13.0 vs. 11.2%, *p* = 0.39).

### 3.2. Plasma Metabolite Concentrations

On day 110 on gestation plasma β-OHB concentrations were higher in dams fed the BD-supplemented diet compared with those on the control diet (0.43 vs. 0.52 μM, respectively, *p* = 0.01) (Table 4). Plasma glucose concentration tended to be higher in dams fed the control diet compared with those fed the BD diet (7.72 vs. 7.50 mM, *p* = 0.09) (Table 4). Plasma NEFA concentrations were lower in dams fed the BD diet (327 vs. 249 μM, respectively, *p* < 0.001) (Table 4). There were no main or interactive effects of parity or diet on plasma insulin concentrations (Table 4). There were no main or interactive effects of parity or diet on plasma β-OHB, NEFA, or glucose concentrations in dam progeny (Table 4).

## 4. Discussion

The major finding from this study was that dietary BD supplementation to both gilts and sows from d90 of gestation to parturition reduced the percentage of piglets born light-for-age and tended to increase individual piglet and total litter weights. Where responses occurred, they appeared to be greater in GP. Dietary BD supplementation increased dam plasma β-OHB concentrations while decreasing fat mobilization as indicated by reduced plasma NEFA in late gestation. The present study also confirmed the established differences in growth performance between GP and SP.

The higher maternal plasma β-OHB concentrations found in dams supplemented with BD were expected due to the ketogenic nature of metabolized BD. Elevated plasma β-OHB concentrations indicated the potential for increased energy sparing in the dam, thus allowing more energy to be diverted towards late gestational growth and energy deposition in the fetus, or in the case of gilts, growth of the dam. Plasma NEFA concentrations were reduced in dams supplemented with BD indicating reduced fat mobilization, since plasma NEFA concentrations are directly related to NEFA turn over and fat mobilization [11]. Furthermore, at weaning, P2 backfat tended to be increased in dams that consumed the BD diet. Although no P2 measurements were taken at d110, there seems to be this effect of reduced fat mobilization throughout the pre-weaning period stemming from d110 reflected in increased P2 backfat at weaning. A reduction in plasma NEFA concentrations due to exogenous ketone supplementation has previously been reported in humans and is most likely attributed to a homeostatic negative feedback mechanism via the PUMA-G receptor [11,12]. This unique co-existence of low NEFA and high β-OHB is likely due to the high affinity of β-OHB to the PUMA-G receptor in adipose tissue with suppresses lipolysis, reducing the plasma NEFA concentrations [12]. Moreover, plasma glucose concentrations tended to be lower in dams supplemented with BD. This effect has been previously associated with exogenous ketone supplementation and was attributed to the ability of ketones to reduce blood glucose by limited hepatic gluconeogenesis and increasing peripheral glucose uptake [12,13]. Dietary BD supplementation of the dam had no effect on β-OHB on concentrations in the progeny.

Piglets born to dams on the BD diet were heavier at birth compared to their control counterparts. There were also less piglets that were light-for-age and therefore at high risk of morbidity and mortality. Low body weight indicates poor productivity and higher chances of pre-weaning mortality in the piglet [1,14,15,16,17]. Therefore, based on the birth weights, BD has the potential to increase pre-weaning survival and productivity. Elevated plasma β-OHB concentrations in the BD-supplemented dams, coupled with increased birth weights, suggest that ketone bodies effectively passed through the placenta and provided an energetic substrate for the neonatal piglet. Although these piglets did not have increased plasma β-OHB concentrations at birth, this may have been due to its rapid metabolism during the initial hours post-farrowing. Such metabolism could provide enough energy for the piglet to rapidly reach the udder and obtain colostrum. Increased NEFA concentrations in piglets have been previously reported as approximately twenty-fold higher than generally observed in growing pigs [11], which is most likely due to increased colostrum consumption. Colostrum has a high triglyceride content, particularly in gilts [10,18,19], and hydrolysis of circulating triglycerides can contribute to elevated NEFA when pigs consume a high fat diet [20]. If glycogen stores were rapidly metabolized in the first 16 h of life, a switch to ketone metabolism may provide supplementary energy for the piglet to rapidly reach the udder and obtain colostrum in piglets born to dams fed the BD diet. While there were no main or interactive effects, BD did not have a significant effect on individual or litter weight at 21 days of age. Therefore, further work is needed to determine whether the effect of BD on birth weight is carried through to weaning and subsequent market weight.

There were differences in mortality within treatment groups in the present study, although the predominant effect was that mortality was greater in GP than in SP. While there was no effect of BD on mortality in the present study, a reduction in mortality of piglets born to dams supplemented with BD has been previously reported [21,22]. For example, two studies reported an increase in fetal hepatic glycogen stores in the piglet from sows fed diets supplemented with BD [22]. The effect is largely attributed to the glucose sparing mechanism of ketones which provide an alternative substrate for oxidation in peripheral tissues in the neonatal pig. However, the present study did not investigate fetal hepatic glycogen levels. With BD supplementation in diets, the provision of an alternative energetic substrate and increased glycogen stores could increase thermoregulatory capacity which is strongly related to piglet survival. Increased thermoregulatory capacity also decreases time to reach the udder, and in turn, increases colostrum and milk consumption. The increased birth weights seen in piglets of the BD diet suggest this mechanism.

## 5. Conclusions

The supplementation of late gestation diets with BD reduced the percentage of small-for-age piglets for both GP and SP and increased birthweight, as indicated by their birth weights. Dietary BD also increased dam plasma β-OHB concentrations and decreased plasma NEFA and glucose concentrations, indicating an energy sparing effect in the dams. However, the effects of BD were not evident at weaning. Further work is required to determine whether the effect of BD on birthweight is carried through subsequent stages of growth, particularly in GP.

## Figures and Tables

**Table 1:** Composition of late gestation diets.

Ingredient (%)	Control	Butanediol (BD)
Wheat	49.8	48.3
Barley	35.0	35.0
Canola meal 38%	6.0	6.0
Meat meal 58%	4.0	4.0
Tallow	2.5	
1,3 Butylene Glycol ^1^		4.0
Limestone	2.0	2.0
Threonine	0.05	0.05
Mineral blend ^2^	0.18	0.18
Vitamin blend ^3^	0.13	0.13
Calculated Composition (DM Basis)		
Energy (MJ DE/kg)	13.8	13.8
Crude protein (%)	14.3	14.2
Crude fat (%)	4.30	3.48
Crude fibre (%)	3.88	3.84
Ash (%)	5.28	5.25
Available lysine (%)	0.71	0.71

^1^ 1,3 Butylene Glycol (Consolidated Chemical Co., Dandenong South, Australia). ^2^ Supplied per kg of diet: Fe 80 mg; Zn 60 mg; Mn 30 mg; Cu 8 mg, as organic trace elements. ^3^ Supplied per kg of diet: vitamin A, 15,000 IU; vitamin D3, 3125 IU; vitamin E, 75 IU; vitamin K, 1 mg; vitamin B1, 1.5 mg; vitamin B2, 5 mg; vitamin B6, 3 mg; vitamin B12, 70 μg; niacin, 20 mg; pantothenic acid, 15 mg; folic acid, 10 mg; vitamin C, 50 mg.

**Table 2:** Farrowing performance of gilts and sows fed a control or a 1,3-Butanediol (BD) diet, and their litter characteristics.

Parity (P)	Gilt		Sow		SED	*p*-Value		
Diet (D)	Control	BD	Control	BD		P	D	P×D
Dam performance								
d90 liveweight (kg)	195	195	239	249	5.0	<0.001	0.15	0.16
d110 liveweight (kg)	208	209	252	260	4.9	<0.001	0.21	0.32
Wean liveweight (kg)	186	186	227	232	5.6	<0.001	0.43	0.50
△ liveweight d90—Wean (kg) ^1^	−8.1	−8.1	−11.2	−14.1	3.7	0.096	0.58	0.59
Wean P2 backfat (mm)	19.2	19.9	19.4	21.2	1.00	0.36	0.082	0.46
△ P2 backfat d90—Wean (mm) ^2^	−1.3	−2.9	−3.2	−3.4	0.76	0.025	0.11	0.20
Litter characteristics at birth								
Born alive	11.8	12.4	12.4	12.1	0.43	0.57	0.67	0.17
Still born	0.43	0.30	0.51	0.42	0.23	0.53	0.48	0.90
Mummified	0.08	0.22	0.17	0.12	0.10	0.97	0.53	0.18

^1^ Change in liveweight from d90 to wean. ^2^ Change in P2 backfat from d90 to wean.

**Table 3:** Growth performance parameters of gilt and sow progeny fed a control or 1,3-Butanediol (BD) diet at 24 h and 21-days of age.

Parity (P)	Gilt		Sow		SED	*p*-Value		
Diet (D)	Control	BD	Control	BD		P	D	P × D
Birth liveweight (*n*)	428	468	455	471				
Individual liveweight (kg)	1.34	1.39	1.51	1.56	0.02	<0.001	0.085	0.98
Total litter (kg)	15.5	17.1	18.5	18.7	0.69	<0.001	0.078	0.15
Intra-litter CV (%)	17.2	17.5	18.4	16.6	0.01	0.89	0.39	0.21
21-day liveweight (*n*)	*327*	*385*	*395*	*411*				
Individual (kg)	5.02	4.98	6.18	6.03	0.01	<0.001	0.064	0.43
Total litter (kg)	47.1	50.6	66.4	62.8	3.10	<0.001	0.83	0.10
Intra-litter CV (%)	20.4	18.5	20.7	21.3	0.17	0.17	0.58	0.30

**Table 4:** Plasma metabolites of gilts and sows and their progeny for the Control and 1,3-Butanediol (BD) diets.

Parity (P)	Gilt		Sow		SED	*p*-Value		
Diet (D)	Control	BD	Control	BD		P	D	P × D
Dam (day 110 of gestation)								
β-OHB (mM)	0.49	0.54	0.45	0.49	0.040	0.96	0.01	0.12
Glucose (mM)	7.72	7.63	7.71	7.36	0.181	0.27	0.09	0.32
Insulin (pM)	409	404	435	433	63.6	0.54	0.94	0.97
NEFA (µM) ^a^	5.75 (314)	5.48 (241)	5.83 (340)	5.54 (256)	0.089	0.28	<0.001	0.86
Progeny (birth)								
β-OHB (mM)	0.42	0.42	0.43	0.36	0.050	0.49	0.35	0.28
Glucose (mM)	8.28	8.47	8.64	8.50	0.209	0.21	0.68	0.27
NEFA (µM) ^a^	6.51 (799)	6.46 (891)	6.32 (662)	6.49 (780)	0.110	0.30	0.53	0.17

^a^ Data were log_e_ transformed before analyses due to heterogeneity of variances. Values in parentheses are back-transformed means.
